# β2 Integrin Regulation of Neutrophil Functional Plasticity and Fate in the Resolution of Inflammation

**DOI:** 10.3389/fimmu.2021.660760

**Published:** 2021-03-30

**Authors:** Meriem Sekheri, Amira Othman, János G. Filep

**Affiliations:** ^1^Department of Pathology and Cell Biology, University of Montreal, Montreal, QC, Canada; ^2^Department of Biomedical Sciences, University of Montreal, Montreal, QC, Canada; ^3^Research Center, Maisonneuve-Rosemont Hospital, Montreal, QC, Canada

**Keywords:** neutrophils, neutrophil trafficking, phagocytosis-induced cell death, apoptosis, NET formation, immunity, resolution of inflammation, Mac-1 (αMβ2)

## Abstract

Neutrophils act as the first line of cellular defense against invading pathogens or tissue injury. Their rapid recruitment into inflamed tissues is critical for the elimination of invading microorganisms and tissue repair, but is also capable of inflicting damage to neighboring tissues. The β_2_ integrins and Mac-1 (CD11b/CD18, α_M_β_2_ or complement receptor 3) in particular, are best known for mediating neutrophil adhesion and transmigration across the endothelium and phagocytosis of microbes. However, Mac-1 has a broad ligand recognition property that contributes to the functional versatility of the neutrophil population far beyond their antimicrobial function. Accumulating evidence over the past decade has demonstrated roles for Mac-1 ligands in regulating reverse neutrophil transmigration, lifespan, phagocytosis-induced cell death, release of neutrophil extracellular traps and efferocytosis, hence extending the traditional β_2_ integrin repertoire in shaping innate and adaptive immune responses. Understanding the functions of β_2_ integrins may partly explain neutrophil heterogeneity and may be instrumental to develop novel therapies specifically targeting Mac-1-mediated pro-resolution actions without compromising immunity. Thus, this review details novel insights on outside-in signaling through β_2_ integrins and neutrophil functional heterogeneity pertinent to the resolution of inflammation.

## Introduction

Neutrophils are the first line of cellular defense against invading pathogens or tissue injury. Rapid recruitment of neutrophils into infected or injured tissues is critical for the elimination of invading microorganisms and tissue repair (1). Ideally, once the pathogens are cleared, cessation of neutrophil recruitment and removal of emigrated neutrophils from the inflamed site will assure timely resolution of inflammation and return to homeostasis (2–4). Aberrant neutrophil accumulation or removal from the inflamed area inflicts damage to the surrounding tissue (2). Indeed, neutrophil-driven tissue injury has been recognized as a common mechanism underlying a wide variety of pathologies, including atherosclerosis, respiratory, autoimmune and neurodegenerative diseases, arthritis, sepsis and cancer (5, 6). Since neutrophils are also involved in the resolution of inflammation (7, 8), the balance between their protective and deleterious actions will likely determine the outcome of the inflammatory response.

The β_2_ integrins LFA-1 (leukocyte function antigen 1, CD11a/CD18) and Mac-1 (CD11b/CD18, α_M_β_2_ or complement receptor 3) are best known for mediating neutrophil adhesion and transmigration across the activated endothelium and phagocytosis of microbes (9–11). Historically, LFA-1 and Mac-1 have been considered pro-inflammatory for reduced expression or function of β_2_ integrins causes rare immunodeficiency syndromes, leukocyte adhesion deficiency syndromes (LAD types I-III), characterized by recurrent infections (12, 13). The binding of Mac-1 and LFA-1 to their endothelial counter-ligand ICAM-1 or matrix components generates survival cues for neutrophils (14, 15). Mac-1 may also contribute to sustained inflammation by enhancing the function of heterologous receptors such as Toll-like receptors and Fcγ receptors through modulating intracellular signaling (16, 17). Accumulating data indicates that Mac-1 can bind a variety of ligands (18). This broad ligand recognition property contributes to the functional versatility of the neutrophil population and shapes innate and adaptive immune responses far beyond their antimicrobial functions. In this review, we will focus on recent advances on outside-in signaling through β_2_ integrins and neutrophil functional heterogeneity during homeostasis and diseases. We also examine how targeting β_2_ integrin signaling could be exploited for facilitating the resolution of inflammation.

## Beta 2 Integrin Activation and Ligand Binding

The β_2_ integrins, composed of a common β_2_ (CD18) subunit complexed with unique α subunits (CD11a-d), are a family of myeloid cell-specific adhesion molecules with LFA-1 (leukocyte function antigen 1, CD11a/CD18) and Mac-1 (CD11b/CD18, α_M_β_2_ or complement receptor 3) being the most studied members. β_2_ integrin ligand binding relies on conformational changes in their ectodomain (19, 20). Ligation of G-protein-coupled receptors or heterologous receptors generates intracellular signals that shift the resting bent/closed β_2_ integrin conformation (low affinity for ligands) to an extended (E+) and then a high-affinity conformation with an “open” headpiece (H+) (canonical “switchblade” model) (19, 21). Spatiotemporal integrin activation is governed by inside-out (i.e. activation of ligand binding function of integrins) and outside-in signaling cascades (i.e. cellular responses evoked by ligand binding to integrins) and involves inhibitory proteins and activator complexes, such as talin, kindlins, cytohesin-1 and integrin-linked kinase, interacting with the cytoplasmic tail of the β subunit (13, 22, 23). The Src kinase-associated phosphoprotein 2 (Skap2), which regulates actin polymerization and binding of talin-1 and kindlin-3 to the β_2_ integrin cytoplasmic domain, is indispensable for β_2_ integrin activation (24). Loss of Skap2 function causes a LAD-like phenotype in mice (24). Mac-1 has two spatially distinct binding sites, the αI-domain and the lectin-like domain (25). The αMI-domain recognizes sequence patterns (consisting of a core of basic residues flanked by hydrophobic residues), rather than specific amino-acid sequence(s) (18) with over 30 structurally unrelated ligands, including ICAM-1, fibrinogen, complement 3b (iC3b), various granule proteins and heparane sulfate (25). The interaction between the αMI-domain and cationic proteins is mediated mostly by hydrophobic contacts independently of divalent cations (26). The lectin-like domain binds β-glucans present in the fungal cell wall (27, 28). **Table 1** lists selected Mac-1 ligands and their main biological actions.

**Table 1:** Selected Mac-1 (CD11b/CD18) ligands and their actions.

Ligands	Species	Effects	Mechanism	References
*Binding site: α_M_I-domain*				
ICAM-1	Human Mouse	Mediates neutrophil adhesion and transmigration	β_2_ integrin conformational changes	(10, 11, 29, 30)
	Human	Limits neutrophil adhesion	High affinity bent conformation of β2 integrins	(31–33)
	Human	↑ Neutrophil lifespan ↓ Apoptosis	↑ Akt, ↑ ERK ↑ Mcl-1	(14, 15, 34, 35)
Fibrinogen	Human	Initiates coagulation		
		↑ Neutrophil lifespan ↓ Apoptosis	↑ Akt, ↑ ERK, ↑ NF-κB	(36)
Plasminogen	Human	Initiates fibrinolysis		
		↑ Neutrophil lifespan ↓ Apoptosis	↑ Akt, ↑ ERK, ↑ NF-κB	(36, 37)
Myeloperoxidase	Mouse	↓ Neutrophil trafficking	Impaired Mac-1 function	(38)
	Mouse	↑ Endothelial cell damage	Transfer of Mac-1-bound myeloperoxidase	(39)
	Human	↑ Neutrophil lifespan ↓ Apoptosis	↑Akt, ↑ERK, ↑ Mcl-1	(40)
	Human Mouse	↑ Myeloperoxidase and elastase release	↑ Akt, ↑ ERK, ↑ NF-κB	(40, 41)
Neutrophil elastase	Human	Reverse transendothelial migration	Elastase-mediated cleavage of JAM-C	(42)
	Zebrafish			(43)
	Mouse			(44–46)
Proteinase 3	Human	Auto-antigen	Disrupts immune silencing	(47–50)
		↓ Efferocytosis	"Don’t eat me" signal (in cooperation with CD16 and CD177)	(47, 51, 52)
LL-37 (Cathelicidin)	Human	↑ Phagocytosis	Opsonizes bacteria	(53)
	Human	Auto-antigen	Psoriasis	(54)
	Mouse	Auto-antigen	Atherosclerosis (?)	(55)
Platelet factor 4	Human	↑ Phagocytosis	Opsonizes bacteria	(56)
C3b (C3b-opsonized bacteria)	Human	↑ Phagocytosis ↑ PICD	↑ ROS, ↑ caspase-3 ↓ Mcl-1	(14, 57, 58)
	Mouse	↑ PICD ↑ Bacterial clearance		(57, 58)
CD40 ligand	Mouse	↑ Leukocyte recruitment ↑ Atherogenesis	Mac-1 as an alternate receptor for CD40L (independent of CD40)	(59)
Dynorphin A	Mouse	↑ Migration ↑ Phagocytosis		(60)
*Binding site: Lectin-like domain*				
Fungus: *A. fumigatus, C. albicans*	Human	↑ NET release	↓ or ↑ ROS, ↑ Syk, ↑ PAD4 (fungus species- dependent)	(61–64)
Immobilized fungal β-glucan	Human	↑ NET release	ROS-independent	(62, 63)
*Binding sites α_M_I-domain and Lectin-like domain*:				
C3b-opsonized tumor cells treated with β–glucan	Mouse	↑ Tumor cell killing	↑ Syk, ↑ PI3K, ↑ Mac-1 toxicity Dual Mac-1 ligation	(28)

C3b, complement 3b; NET, neutrophil extracellular trap; PAD4, protein-arginine deiminase type 4; PICD, phagocytosis-induced cell death; ROS, reactive oxygen species.

## Limiting Neutrophil Trafficking Into Tissues

Neutrophils exit the circulation at the sites of inflammation through the classical adhesion cascade (10). The molecular mechanisms mediating and governing this multistep process as well as organ-specific differences have been described in detail (10, 29, 30, 65). In general, β_2_ integrins play vital roles in neutrophil arrest on the activated endothelium under flow (10), transmigration through endothelial cells (66), chemotaxis (67) and neutrophil swarming (68). Counter-ligand-specific binding forces of LFA-1 and Mac-1 imply diverse roles for β2 integrins in neutrophil recruitment (69) and determine the direction of neutrophil migration along the activated endothelium (70). Fully activated E^+^H^+^ β_2_ integrins bind ICAM-1 expressed on the opposing cells in *trans* and arrest neutrophil rolling (31). Studies with human neutrophils in microfluidic chambers identified high-affinity, bent conformation (E^-^H^+^) β_2_ integrins, which face each other to form oriented nanoclusters (32) and bind ICAM-1 in *cis* to inhibit neutrophil rolling and consequently neutrophil adhesion to the endothelium (31). Activated β_2_ integrins may also restrict neutrophil recruitment during acute bacterial infections, for pharmacological inhibition of high-affinity β_2_ integrins or genetic deletion of talin-1 or kindlin-3 was found to enhance neutrophil trafficking with modest impairment of phagocytosis during *Pseudomonas aeruginosa*-pneumonia in mice (71). Another potential inhibitory signal is the interaction of the αI-domain of Mac-1 in the bent state with the sialylated ectodomain of the IgG receptor FcγRIIA in *cis*, leading to reduced FcγRIIA affinity to IgG and subsequently decreased neutrophil recruitment to immune complexes deposited in the vessel wall (33). Disruption of this interaction may increase neutrophil recruitment in autoimmune diseases.

Neutrophils from myeloperoxidase knockout mice display increased surface expression of Mac-1 and a pro-migratory phenotype in a murine model of ischemia-reperfusion-induced liver damage (38). Hence, myeloperoxidase may impair Mac-1 function and subsequently limit neutrophil trafficking into ischemic tissues. Neutrophil-derived myeloperoxidase was reported to protect mice from endotoxin-induced inflammation and mortality (72), though the involvement of β_2_ integrins in these actions remains to be investigated. On the other side, cell contact-dependent, Mac-1-mediated transfer of myeloperoxidase from neutrophils to endothelial cells can disrupt normal endothelial function (39), leading to endothelial inflammation that underlies atherosclerosis and vasculitis. Following neutrophil adhesion to the endothelium, gelatinase granules translocate to the cell surface and externalize the phospholipid-binding protein annexin A1 (73). Annexin A1 promotes the detachment of adhering leukocytes presumably through inhibiting CCL5-induced switch in β_2_ integrin conformation, and reducing α_4_β_1_ integrin clustering and activation (74, 75). Hence, annexin A1 may function as an endogenous stop signal for neutrophil trafficking (76).

## Reverse Transendothelial Migration

**Figure 1:**
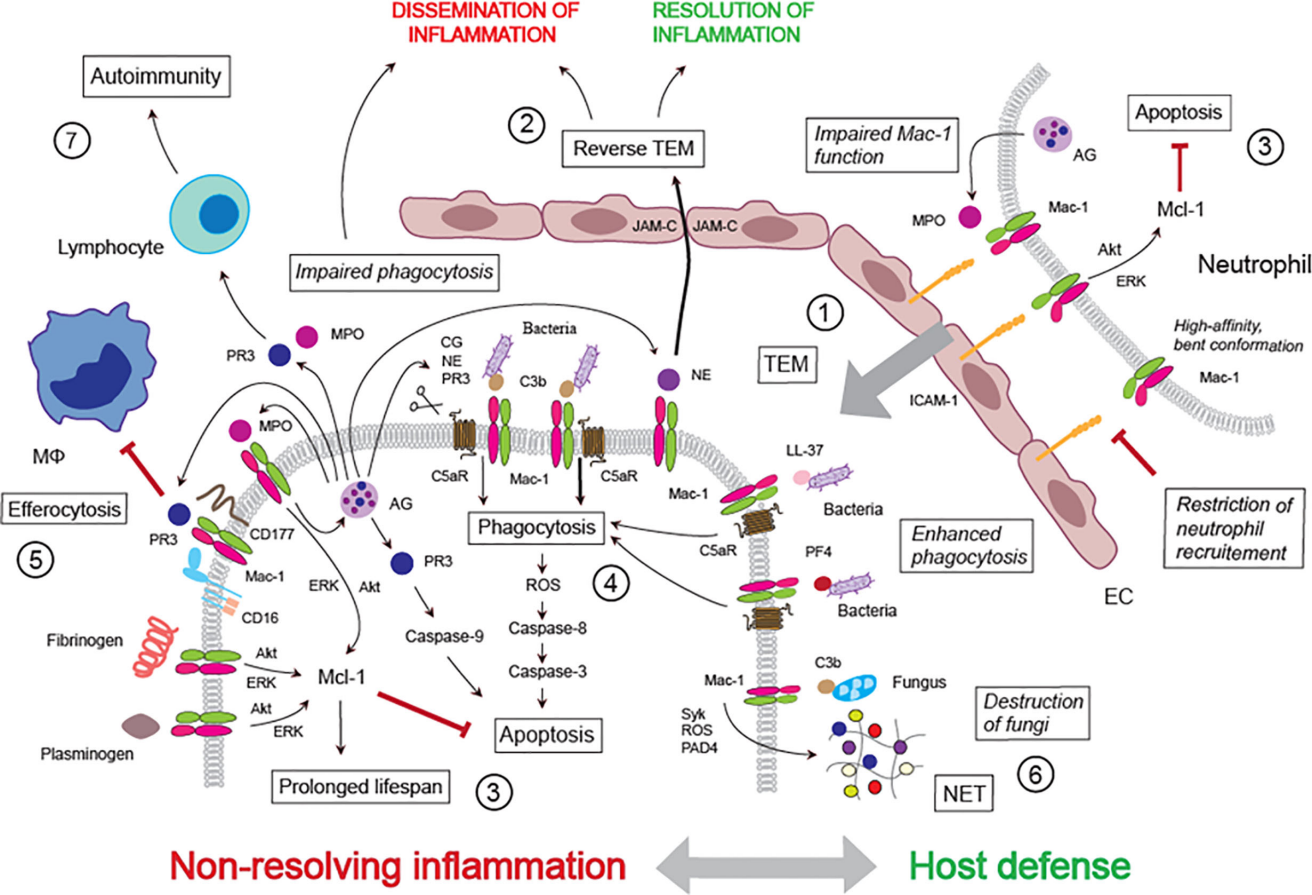
Mac-1 ligand repertoire shapes host defense and non-resolving inflammation. *① Transendothelial migration*: Mac-1, together with LFA-1, mediates neutrophil adherence to the activated endothelium and transmigration. Conformational changes in Mac-1 (high affinity, bent conformation) and MPO impairment of Mac-1 function may limit neutrophil trafficking. *② Reverse TEM*: Mac-1-bound NE direct neutrophil reverse TEM through binding to and cleaving JAM-C. *③ Neutrophil lifespan*: Ligation of Mac-1 with ICAM-1, fibrinogen, plasminogen or MPO generates survival signals for neutrophils through delaying constitutive apoptosis. MPO induces MPO release from the azurophilic granule, thereby forming a feed-forward loop. *④ Phagocytosis:* Phagocytosis of complement C3b-opsonized bacteria induces PICD followed by efferocytosis. Cleavage of C5aR (CD88) by NE, PR3 or cathepsin G (released from the azurophilic granule) alters the Mac-1/C5aR ratio, impairs phagocytosis, bacterial clearance and PICD. *⑤ Inhibition of efferocytosis*: PR3 bound to Mac-1(in association with CD16 and CD177) inhibits efferocytosis. *⑥ NET release*: C3b-opsonized fungus or immobilized fungal β-glucan, which cannot be phagocytosed, evokes release of NET, leading to extracellular killing of the pathogen. *⑦ Autoimmunity*: PR3 and MPO (presented by Mac-1 and/or NET) may induce autoimmunity.

In addition to moving from the vascular lumen to the extravascular tissue, neutrophils also exhibit reverse motility through the endothelium, known as reverse transendothelial migration (TEM) both *in vitro* (42) and *in vivo* (44, 45). This neutrophil reverse TEM response is relatively prevalent under conditions of ischemia-reperfusion injury, which is associated with reduced expression of junctional adhesion molecule C (JAM-C) at endothelial cell junctions (45, 46). Pharmacological blockade or genetic deletion of JAM-C enhances the frequency of neutrophil reverse TEM in mouse cremaster venules (45). Under ischemia-reperfusion, locally generated LTB_4_, likely produced by the neutrophils themselves (68), induces elastase release from neutrophils through the LTB_4_ receptor BLT1 (44). Activated Mac-1 binds neutrophil elastase (77) and JAM-C (78), thereby acting as a molecular “bridge” to facilitate elastase-mediated cleavage of JAM-C and consequently reverse TEM (44) (**Figure 1**). The importance of Mac-1-bound elastase is further highlighted by the failure of exogenous neutrophil elastase to cleave JAM-C (44). Reversely migrated neutrophils display a phenotype (ICAM-1^high^, CXCR1^low^) distinct from tissue-resident or circulating neutrophils and increased capacity to produce superoxide (42, 45). At present, the functional implications of neutrophils undergoing reverse TEM remain unclear. Reverse TEM might facilitate the removal of neutrophils from inflamed tissues, thereby promoting the resolution of inflammation (43, 79). Alternatively, re-entry of a small subset of activated neutrophils into the blood circulation could contribute to spreading a local inflammatory response, ultimately leading to distant organ damage (44, 45). This notion is supported by the association between the percentage of ICAM-1^high^ neutrophils and the severity of lung inflammation in the mouse cremaster ischemia-reperfusion model (45).

## Extending Neutrophil Lifespan and Suppression of Apoptosis

Circulating neutrophils have a short lifespan (80, 81), though some reports estimated that their lifespan to be 5.4 days (82). Neutrophils have increased, albeit variable lifetimes upon activation and in healthy and inflamed tissues (4, 6, 83, 84). Blood neutrophils die by constitutive apoptosis. This cell death program renders neutrophils unresponsive to extracellular stimuli and ensures their timely removal from the inflammatory sites by macrophages *via* efferocytosis, thereby limiting their potentially harmful actions to the host (2, 85, 86). Extended neutrophil lifespan through suppressed apoptosis is observed in patients with chronic inflammation, for example, acute coronary syndrome (87), asthma (88) or sepsis (89), and is associated with increased disease severity. Consistently, studies in experimental models documented that delaying neutrophil apoptosis can adversely affect the outcome of inflammation (40, 90, 91).

During transendothelial migration and at sites of inflammation, neutrophils receive pro-survival cues that extend their lifespan by delaying intrinsic apoptosis (4, 85, 86). Neutrophil adherence to the Mac-1 endothelial counter-ligand ICAM-1induces activation of the PI3k/Akt and MAPK/ERK pathways (34, 35), leading to suppression of caspase-3 activity through preserving the anti-apoptotic protein Mcl-1, a key regulator of neutrophil survival (92). Suppression of apoptosis by the Mac-1 ligands fibrinogen and plasminogen also depends on signaling through Akt and ERK as well as activation of NF-κB (36). Engagement of both Mac-1 subunits with soluble ligands is essential for the generation of pro-survival cues, whereas adhesion *per se* is not a prerequisite (37). Another ligand for Mac-1 is myeloperoxidase, a granule protein implicated in pathogen killing and inflicting tissue damage (93–96). Myeloperoxidase binding to Mac-1on human neutrophils leads to activation of the PI3K/Akt, p38 MAPK, MAPK/ERK and NF-κB pathways (40, 41) and rescues neutrophil from apoptosis (40). Myeloperoxidase upregulates Mac-1 expression and induces myeloperoxidase release from the primary granules (40, 41), thereby forming an autocrine/paracrine feed-forward loop to amplify the inflammatory response (40) (**Figure 1**). Increased plasma myeloperoxidase levels were detected in patients with acute coronary syndromes or sepsis and were associated with disease severity (41). Dissociation of myeloperoxidase into monomers with diminished biological activities may represent a mechanism to limit neutrophil responses to this protein (97).

## Phagocytosis and Phagocytosis-Induced Cell Death

In contrast to Mac-1 ligation-generated survival signals, outside-in signaling through Mac-1 could also generate pro-apoptosis cues. Thus, phagocytosis of complement C3b-opsonized bacteria or necrotic cells accelerates neutrophil apoptosis, also known as phagocytosis-induced cell death (PICD) (98, 99). The antibiotic peptide LL-37 and platelet factor 4 were also reported to opsonize bacteria and promote Mac-1-mediated phagocytosis (53, 56). Higher levels of Mac-1 expression on neutrophils from female vs. male mice may partly explain an innate sex bias in neutrophil bactericidal killing (100). Phagocytosis is initiated by lateral clustering of Mac-1 (101) and governed by a delicate balance between Mac-1 and the complement C5a receptor (C5aR or CD88) (102, 103). Mac-1-mediated phagocytosis evokes ROS formation through activation of NADPH oxidase, which is thought to mediate bacterial killing in the phagolysosomes (94). ROS, presumably hydroxyl radicals and H_2_O_2_, activate caspase-8 and caspase-3, thereby countering survival signals generated by ligation of Mac-1and promoting PICD (4, 14, 98). Release of the granule enzymes, neutrophil elastase, proteinase 3 and cathepsin G can impair phagocytosis by cleaving C5aR, though their involvement appears to be context-dependent (57, 104) (**Figure 1**). For example, TLR9 activation augments Mac-1 expression and reduces C5aR expression through inducing the release of neutrophil elastase and proteinase 3, resulting in defective phagocytosis in human neutrophils and prolongation of lung injury in mice (57). Reduced neutrophil C5aR expression is a common finding in patients with sepsis (105, 106) and may explain neutrophil unresponsiveness to C5a in sepsis (103).

## Modulation of Efferocytosis

Detection and prompt disposal of apoptotic cells, including neutrophils, generally promote an anti-inflammatory pro-resolution response at the tissue level and immunological tolerance. The molecular mechanisms include numerous “find-me” and “eat-me” signals that underpin the recognition and subsequent phagocytosis of apoptotic cells by macrophages and dendritic cells (107). Intriguingly, proteinase 3 is expressed on the plasma membrane in association with its partners Mac-1, FcγRIIIb (CD16) and CD177 at a very early stage of apoptosis (51, 108) and functions as a “don’t eat me” signal that delays uptake of apoptotic neutrophils (52) (**Figure 1**). Neutrophils lacking CD177, the putative receptor for proteinase 3, express membrane proteinase 3 and respond to proteinase 3-ANCA, suggesting a critical role for Mac-1 and/or FcγRIIIb (47). However, further studies are required to establish how Mac-1 could modulate efferocytosis and contribute to the pathogenesis of ANCA-associated vasculitides. Mac-1 was also reported to support macrophage fusion, leading to the formation of multinucleated giant cells in the inflamed mouse peritoneum (109). The function of these cells remains to be investigated.

## Induction of Rapid NET Release

Neutrophils can release extracellular traps (NET) to immobilize and kill harmful bacterial, fungal and viral pathogens in the extracellular space when phagocytosis is not feasible (110–112). The classical pathway of NET extrusion involves activation of NADPH oxidase *via* the Raf-MEK-ERK and p38 MAPK pathways, myeloperoxidase- and elastase-mediated cleavage of histones and protein-arginine deiminase 4 (PAD4)-mediated chromatin decondensation, eventually leading to extrusion of a DNA scaffold studded with citrullinated histones and cytotoxic granular proteins (113, 114). A more rapid or “vital” NET release occurs in response to *Staphylococcus aureus*, *Candida albicans*, *Aspergillus fumigatus* and Leishmania promastigotes independently of ROS in the presence of matrix and without compromising neutrophil viability (61, 115, 116) or by selective extrusion of mitochondrial DNA (117). Mac-1 recognition of Candida hyphae, the invasive filamentous forms of *C. albicans* that are too large to be phagocytosed, or immobilized fungal β-glucan triggers rapid NET release and initiates respiratory burst, which is then suppressed by binding of Mac-1 to the extracellular matrix (62, 63) (**Figure 1**). Mac-1 also triggers NETosis to Aspergillus living conidia through ROS generation downstream to activation of the Src kinase Syk and PI3k-δ, but independently of PAD4 (64). Platelet binding to neutrophils ensuing NET release is mediated either by LFA1 along liver sinusoid in sepsis (118) or Mac-1 along the vascular endothelium during sterile lung injury in mice (119). Hence, it is plausible that the context of NET-inducing stimuli would activate different signaling pathways for NET extrusion. Similar to neutrophil recruitment, a crosstalk between kindlin-3 and β_2_ integrins is required for NET release in mice (120). Of note, yeast β-glucan was reported to enhance killing of iC3b-opsonized tumor cells through activation of the Syk-PI3K signaling pathway, indicating dual Mac-1 ligation (28) (**Table 1**). Whether the cytotoxic action involves NET formation remains to be explored.

## Autoimmunity

Many neutrophil granule proteins are recognized self-antigens in autoimmunity. Myeloperoxidase and proteinase 3 are target antigens in different forms of anti-neutrophil cytoplasmic antibody (ANCA)-associated vasculitides (48, 49), whereas the antimicrobial protein LL-37 is an autoantigen in psoriasis (54). Externalization of these molecules, together with other well-known antigens, such as double-stranded DNA and histones, through aberrant NET formation has been implicated in triggering a systemic autoimmune response in susceptible individuals (49). Myeloperoxidase might trigger autoimmunity during uncontrolled inflammation in mice (121), though it is unclear whether this involves β_2_ integrins and/or NET formation. Proteinase 3 was found to bind directly to Mac-1 (51) or form a complex with the glycosylphosphatidylinositol (GPI)-anchored neutrophil-specific receptor NB1 (CD177) expressed on the surface of a subpopulation of human neutrophils (122) (**Figure 1**). While surface plasmon resonance analysis indicated direct interaction of NB1 with both LFA1 and Mac-1, only Mac-1 functions as an adaptor for NB1-mediated proteinase 3-ANCA-induced neutrophil activation (123). Proteinase 3 expressed on the surface of apoptotic neutrophils disrupts immune silencing associated with efferocytosis through plasmocytoid dendritic cell-driven generation of Th9/Th2 cells and Th17 response, consistent with promoting systemic necrotizing vasculitis (50). Recent data identified the cathelicidin protein CRAMP (a truncated form of the mouse homolog of hCAP18) as a potential auto-antigen in ApoE-deficient mice (55). Although LL-37 was found to bind to Mac-1 (53), the relevance of this interaction to atherosclerosis remains to be investigated.

## Therapeutic Targeting β2 Integrins to Promote Resolution of Inflammation

In the light of their functional significance in shaping neutrophil responses, β_2_ integrins appear to be attractive therapeutic targets. However, prolonged global blockade of β_2_ integrins may have limited usefulness because of the potential of development of LAD-like symptoms. Attractive alternative approaches may be targeting β_2_ integrin conformation or ligand-specific signaling circuits by specialized pro-resolving mediators (SPMs) without compromising the ability of neutrophils to contain the microbial invasion.

The currently available drugs (monoclonal antibodies or small molecules) inhibit the ligand-binding site and block a broad repertoire of β_2_ integrin functionality (124). Although the beneficial actions of genetic deletion and pharmacological blockade of β_2_ integrins have been documented in a number of neutrophil-driven inflammatory models (6, 29, 124), conventional anti-β_2_ integrin blockade lacks specificity and inhibits phagocytosis, promotes apoptosis, and potentiates bacteremia and bacterial sepsis (125). Development of the anti-M7 monoclonal antibody that specifically inhibits Mac-1 interaction with its ligand CD40L without interfering with other β_2_ integrin ligands (59, 125) opens a new avenue for ligand-targeted anti-Mac-1 therapy. An alternative strategy will be developing allosteric inhibitors that stabilize β_2_ integrins in the high affinity bent conformation to block neutrophil adherence (31, 71) or to prevent the deleterious effects of immune complex-evoked neutrophil accumulation (33). This might be achieved by selectively targeting discrete glycan motifs present on Mac-1 (126). Thus, plant lectins were shown to reduce Mac-1-mediated adhesion, trans-epithelial migration and ROS production, while enhancing phagocytosis and neutrophil apoptosis (126). Intriguingly, the activation of Mac-1 with the small molecule agonists leukadherins was reported to reduce leukocyte trafficking, arterial narrowing and renal dysfunction, while increasing leukocyte adherence to the endothelium in murine models (127). Leukadherin-1 promotes macrophage polarization toward a pro-inflammatory phenotype through activating microRNA Let7a, thereby driving anti-tumor immunity (128).

SPMs include protein and lipid mediators that are mobilized and/or synthesized during the resolution phase of inflammation. For example, annexin A1 is mobilized from the cytoplasm pool to the cell surface and signals through the lipoxin A_4_/formyl-peptide receptor 2 (ALX/FPR2) to induce detachment of adhered neutrophils (74, 129). The family of lipid SPMs consists of lipoxins, resolvins, protectins and maresins (3, 130, 131). These lipids act through specific receptors and exhibit cell-specific properties, however, their primary targets are myeloid cells (3, 132). In general, lipid SPMs prevent up-regulation of Mac-1 expression and inhibit β_2_-integrin-mediated neutrophil adhesion, transendothelial migration and consequently tissue accumulation [signaling pathways and networks are mapped into the searchable Atlas of Inflammation Resolution (133)]. For example, lipoxin A_4_ mobilizes annexin A1 to form an endogenous anti-inflammation loop to limit neutrophil trafficking into inflammatory loci (134). Aspirin triggered 15-epi-lipoxin A_4_, acting through ALX/FPR2, disrupts the myeloperoxidase-centered self-amplifying loop and redirects neutrophil to apoptosis (58), and enhances phagocytosis of bacteria by restoring the balance between Mac-1 and C5aR expression in human neutrophils (57). Consistently, 15-lipoxin A_4_ accelerates the resolution of inflammation in mouse models of myeloperoxidase (58) or *E. coli*-induced acute lung injury (57).

## Conclusions

Unlike other integrins, the β_2_ integrin Mac-1 has two spatially distinct binding sites and exhibits broad ligand recognition specificity and numerous neutrophil responses. A novel aspect of Mac-1 bioactivity is that its conformations and ligands contribute to neutrophil functional plasticity and heterogeneity. The classical view of β_2_ integrins does not accommodate all aspects of their role in neutrophil biology. Their role in regulating neutrophil reverse transmigration, lifespan, phagocytosis-induced cell death, NET formation and efferocytosis extend the β_2_ integrin repertoire in shaping innate and adaptive immunity and may partly explain neutrophil heterogeneity. Understanding the functions of β_2_ integrins may be instrumental to develop novel therapies specifically targeting pro-resolution actions without compromising immunity.

## Author Contributions

MS, AO, and JF conceived, designed, and wrote the manuscript. All authors contributed to the article and approved the submitted version.

## Funding

This study was supported by grants from the Canadian Institutes of Health Research (MOP-97742 and MOP-102619) (to JF).

## Conflict of Interest

The authors declare that the research was conducted in the absence of any commercial or financial relationships that could be construed as a potential conflict of interest.

## Abbreviations

AG, azurophilic granule; C5aR, complement 5a receptor; CG, cathepsin G; EC, endothelial cell; JAM-C, junctional adhesion molecule-C; MΦ, macrophage; MPO, myeloperoxidase; NE, neutrophil elastase; NET, neutrophil extracellular traps; PICD, phagocytosis-induced cell death; PR3, proteinase 3; TEM, transendothelial migration.
